# Mentalization and training in psychiatry: protective factors against burnout. Evidence from a multicenter controlled longitudinal study

**DOI:** 10.1192/j.eurpsy.2026.10959

**Published:** 2026-07-31

**Authors:** L. Tarchi, E. Cassioli, G. Castellini, V. Ricca, A. Fiorillo

**Affiliations:** 1 Health Sciences, University of Florence, Florence; 2 University of Campania “Luigi Vanvitelli”, Caserta, Italy

## Abstract

**Introduction:**

According to mentalization theory, personal and interpersonal skills can be shaped by early adverse experiences and coping strategies, thus playing a key role in mitigating burnout during training. In this study, the interaction between these variables was tested in a multicenter longitudinal survey involving various Italian academic institutions.

**Objectives:**

The primary objective was to investigate protective and risk factors for the development of burnout in psychiatry trainees. Secondary objectives included comparing burnout trajectories among psychiatry residents and those in other medical disciplines, identifying psychiatry -specific factors versus general training longitudinal trajectories, and analyzing the dynamic development of personal/interpersonal skills during psychiatry training.

**Methods:**

A cohort of 1,803 medical residents (including 1,131 psychiatry residents) was assessed for emotional regulation, interpersonal skills, coping strategies, and burnout dimensions using the Difficulties in Emotion Regulation Scale (DERS); the Interpersonal Competence Questionnaire (ICQ); the Maslach Burnout Inventory (MBI). The assessment was repeated after one year of follow-up to examine longitudinal trends. Longitudinal mixed models and bivariate latent change models were used to examine burnout trajectories and related factors.

**Results:**

An increase in burnout was observed in the first two years of specialization. This increase in burnout over the years was lower among psychiatry residents than among other medical residents. Psychiatry residents had a lower increase in burnout over time thanks to greater development of interpersonal skills and coping strategies during their training. In all residents, greater development of burnout was associated with insecure attachment, emotional dysregulation, maladaptive coping, and poorer interpersonal skills. Regular supervision by a supervisor protected against increased burnout. The bivariate latent change model showed that greater development of emotional regulation skills during specialization was associated with a lower increase in burnout.

**Image 1:**
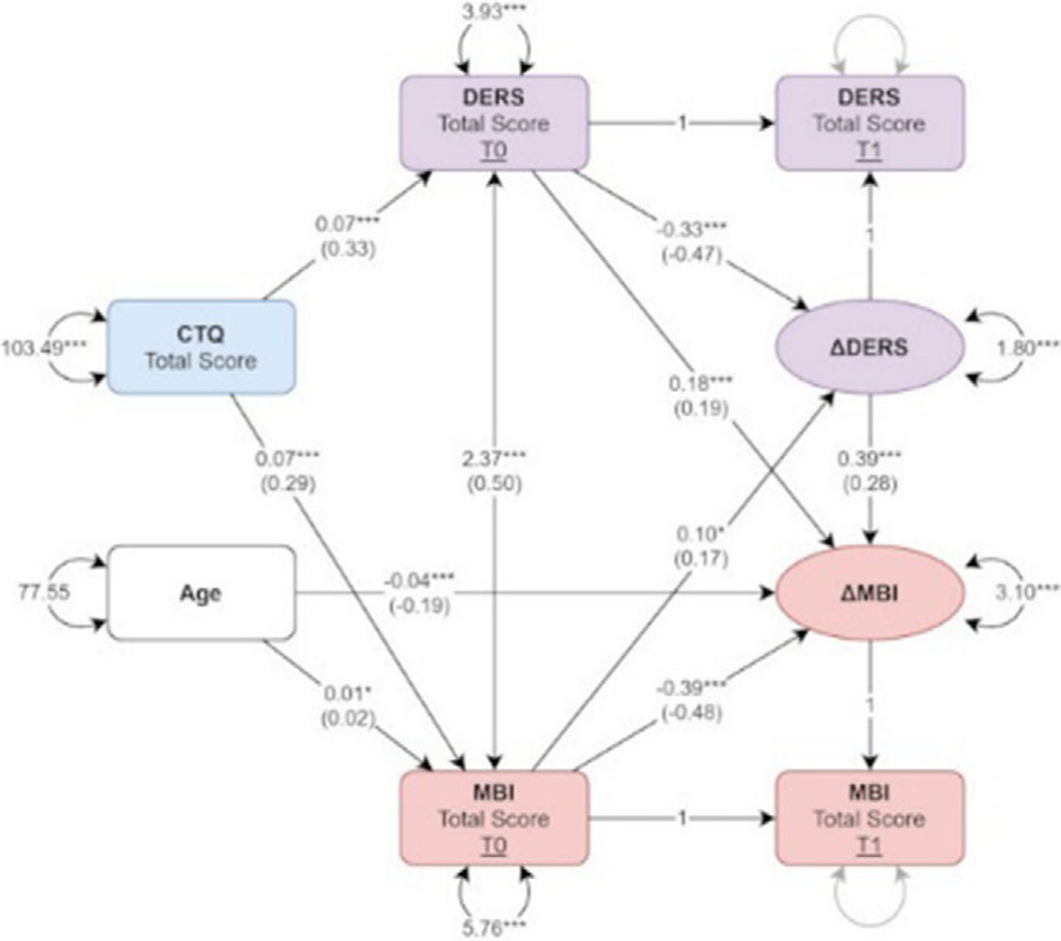
Image 1: Long description.

**Conclusions:**

Mentalization skills can mitigate the development of burnout. Training in psychiatry emerged as a protective factor against burnout, as it was observed as reinforcing specific protective factors. Frequent structured supervision was observed as promoting both professional development and emotional resilience for medical residents in general and psychiatry trainees in particular. The “supervisor” may then be considered as a “secure base” for trainees, allowing residents to remodel and enhance their personal characteristics during training. Training - in this context of mutual trust - was, in fact, associated with an improvement in mentalization skills.

**Disclosure of Interest:**

None Declared

## Long descriptions

### Image 1: Long description

A path diagram with rectangular and oval nodes connected by single-headed arrows (paths) and double-headed arrows (covariances).

On the far left, two independent variables are stacked vertically:

- C T Q Total Score (blue rectangle) with a self-covariance of 103.49***.

- Age (white rectangle) with a self-covariance of 77.55.

In the center column, baseline measurements at T 0 are shown:

- D E R S Total Score T 0 (purple rectangle) at the top, with a self-covariance of 3.93***.

- M B I Total Score T 0 (red rectangle) at the bottom, with a self-covariance of 5.76***.

- A vertical double-headed arrow connects these two with a covariance of 2.37*** (0.50).

On the right side, change scores and T 1 measurements are shown:

- Delta D E R S (purple oval) with a self-covariance of 1.80***.

- Delta M B I (red oval) with a self-covariance of 3.10***.

- D E R S Total Score T 1 (purple rectangle) at the top right.

- M B I Total Score T 1 (red rectangle) at the bottom right.

Key paths include:

- C T Q to D E R S T 0: 0.07*** (0.33).

- C T Q to M B I T 0: 0.07*** (0.29).

- Age to Delta M B I: -0.04*** (-0.19).

- Age to M B I T 0: 0.01* (0.02).

- D E R S T 0 to Delta D E R S: -0.33*** (-0.47).

- D E R S T 0 to Delta M B I: 0.18*** (0.19).

- M B I T 0 to Delta D E R S: 0.10* (0.17).

- M B I T 0 to Delta M B I: -0.39*** (-0.48).

- Fixed paths of 1 connect T 0 scores to T 1 scores and Delta scores to T 1 scores.

Navigate back to Image 1:.

